# PACAP in hypothalamic regulation of sleep and circadian rhythm: importance for headache

**DOI:** 10.1186/s10194-018-0844-4

**Published:** 2018-03-05

**Authors:** Philip R. Holland, Mads Barloese, Jan Fahrenkrug

**Affiliations:** 1 Department of Basic and Clinical Neuroscience, Headache Group, Institute of Psychiatry, Psychology and Neuroscience, King’s College London, London, UK; 2 Department of Clinical Physiology, Nuclear Medicine and PET, 70590 Rigshospitalet, Copenhagen, Denmark; 3 Department of Clinical Biochemistry, Faculty of Health and Medical Sciences, Bispebjerg Hospital, University of Copenhagen, Copenhagen, Denmark

**Keywords:** Migraine, Cluster headache, Circadian, Circannual, Hypothalamus, Pituitary adenylate cyclase-activating peptide

## Abstract

The interaction between sleep and primary headaches has gained considerable interest due to their strong, bidirectional, clinical relationship. Several primary headaches demonstrate either a circadian/circannual rhythmicity in attack onset or are directly associated with sleep itself. Migraine and cluster headache both show distinct attack patterns and while the underlying mechanisms of this circadian variation in attack onset remain to be fully explored, recent evidence points to clear physiological, anatomical and genetic points of convergence. The hypothalamus has emerged as a key brain area in several headache disorders including migraine and cluster headache. It is involved in homeostatic regulation, including pain processing and sleep regulation, enabling appropriate physiological responses to diverse stimuli. It is also a key integrator of circadian entrainment to light, in part regulated by pituitary adenylate cyclase-activating peptide (PACAP). With its established role in experimental headache research the peptide has been extensively studied in relation to headache in both humans and animals, however, there are only few studies investigating its effect on sleep in humans. Given its prominent role in circadian entrainment, established in preclinical research, and the ability of exogenous PACAP to trigger attacks experimentally, further research is very much warranted. The current review will focus on the role of the hypothalamus in the regulation of sleep-wake and circadian rhythms and provide suggestions for the future direction of such research, with a particular focus on PACAP.

## Background

Primary headache disorders represent a group of diverse neurological attack forms that present with varying intensity, duration, frequency and associated symptoms [1]. Despite these underlying differences the hypothalamus has emerged as a critical component of several attack forms, including migraine [2–5] and cluster headache [6–8]. The hypothalamus is a key regulator of homeostatic mechanisms including sleep-wake cycles that are under circadian regulation [9]. Given the circadian and circannual nature of several attack forms [10–12], the clinical association with sleep disturbances [13, 14] and neuroimaging data supporting abnormal hypothalamic activation in several primary headache disorders [2, 4–6, 8, 15] there is an unmet need to develop novel mechanistic insight that may herald novel therapeutic strategies. In particular pituitary adenylate cyclase-activating peptide (PACAP) has emerged as a key neuropeptide involved in migraines and, as a parasympathetic and hypothalamic signaling molecule, that may be involved in cluster headache. PACAP is known to trigger migraine [16, 17] in susceptible individuals, plays a key role in hypothalamic circadian entrainment to light [18] and is the subject of significant interest as a potential therapeutic target for migraine and cluster headache [19, 20]. As such, the current review will focus on the potential regulation of sleep and circadian mechanisms in primary headache disorders with a particular focus on the regulation and future therapeutic potential of modulating PACAP signaling.

### Introduction

The ability to adapt to external environmental conditions is a fundamental principle for the survival of an organism. As such several systems have evolved that permit homeostatic regulation to internal and external cues, facilitating appropriate physiological responses. These are most evident in the daily regulation of sleep-wake cycles with its circa 24-h rhythmicity (circadian), but also include circannual (yearly), infradian (> day) and ultradian (< day, but > one hour) rhythms. Sleep itself is generally dissected into wakefulness, non-rapid eye movement (NREM), and paradoxical or rapid eye movement (REM) sleep. Encephalographically, REM sleep and wakefulness are indistinguishable with fast, low amplitude, desynchronized oscillations, whereas NREM sleep stages I-III are characterized by increasingly lower frequencies of synchronized cortical activity. The different stages of sleep are precisely regulated, complex mechanisms ensuring their consolidation at specific times (for review see [21]), timely progression and avoidance of intermediary stages.

**Fig. 1:**
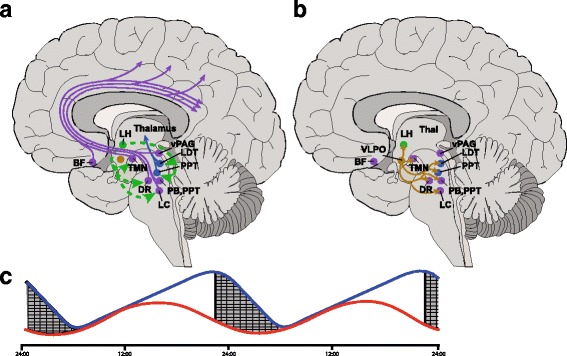
Mechanisms regulating sleep wake modulation. **a**. Orexinergic neurons originating in the lateral hypothalamus (LH; Green) send excitatory projections to several brainstem nuclei that act to promote arousal. Ascending monoaminergic projections (purple) from the noradrenergic locus coeruleus (LC), glutamaterigic parabrachial (PB) and pedunculopontine (PPT), serotoninergic dorsal raphe (DR), dopaminergic ventral periaqueductal grey (vPAG), tuberomammillary nuceus (TMN) and GABAergic and cholinergic neurons in the basal forebrain (BF) diffusely innervate the cerebral cortex to promote arousal. There are also cholinergic projections (Blue) from the laterodorsal tegmental nuclei (LDT) and PPT nuclei that project to the thalamus to promote arousal. **b**. GABAergic ventrolateral preoptic (VLPO) neurons (Brown) act to inhibit the majority of the arousal nuclei, including LH orexinergic neurons to promote sleep. **c**. Homeostatic sleep pressure (Blue line) increases through wakefulness, likely via the accumulation of endogenous somnogens such as adenosine that excites VLPO neurons to promote sleep. This is combined with circadian sleep regulation (Red line) to create a balanced sleep wake cycle that is entrained to external environmental conditions. The circadian component is in part dependent on pituitary adenylate cyclase-activating peptide signalling within the hypothalamic suprachiasmatic nucleus as demonstrated by preclinical research

While a complete understanding of the function of sleep remains to be fully characterized it clearly has a restorative effect on the brain [22]. It is proposed to be regulated by at least two divergent mechanisms including circadian and homeostatic sleep pressure. This elegant regulatory mechanism allows the body to respond to “sleep need” via the accumulation of an endogenous somnogens (e.g. Adenosine) on the background of a circadian influence that entrains sleep-wake cycles to external cues such as seasonal light-dark patterns, for review see [9]. The neuroanatomical basis for sleep was initially postulated in response to a wave of “encephalitis lethargica” with the neurologist Von Economo detailing the presence of lesions in the border of the midbrain and diencephalon responsible for this excessive sleepiness [23] and thus forming the basis for our current understanding of arousal networks (see Fig. 1). Complimentary observations in patients presenting with insomnia highlighted lesions within the lateral hypothalamic area, with subsequent studies identifying specific cell groups including the ventrolateral preoptic area (VLPO) that act to promote sleep [24] and inhibit arousal networks [25]. A further seismic step in our understanding of the regulation of sleep-wake cycles came with the proposal of a “flip-flop” switch; whereby hypothalamic orexinergic synthesizing neurons act to reinforce the ascending arousal networks during wakefulness and are reciprocally inhibited in conjunction with the ascending arousal nuclei by the VLPO during sleep [26]. The importance of these neurons in the regulation of arousal is evident in the devastating consequences their loss has on patients suffering from narcolepsy [27].

Given the complex clinical relationship between circadian/sleep regulation and headache, their shared physiological and neuroanatomical basis (see Fig. 1 and reviewed in [9, 28]), the emerging role for the hypothalamus in the regulation of migraine and cluster headache-relevant homeostatic regulation (see [9, 28]) and the emergence of pituitary adenylate cyclase-activating peptide (PACAP) as a key neuropeptide in the regulation of migraine biology [20]. The current review will focus on the role of the hypothalamus in the regulation of sleep-wake and circadian rhythms, with a particular focus on PACAP.

### PACAP

A detailed description of PACAP pharmacology is discussed elsewhere in this special issue and in several recent reviews including [29]. Herein we provide a brief review for orientation purposes. PACAP is widely distributed throughout the peripheral and central nervous system [30]. It occurs in two forms, PACAP-38 and PACAP-27 that are cleaved from the same preproPACAP protein. It is closely related to several neuropeptides including vasoactive intestinal peptide (VIP) and peptide histadine methionine. Interestingly, VIP induces a similar headache [31] to PACAP [32] in healthy volunteers, but fails to induce a delayed migraine-like attack [31, 33]. PACAP and VIP share relatively equal affinity for the VPAC_1_ and VPAC_2_ receptors, whereas PACAP shows a greater affinity for the PAC_1_ receptor (for review see [34]). As such, despite sharing similar signalling mechanisms the PAC_1_ receptor has emerged as the first PACAP receptor to be targeted clinically for migraines [19]. This is supported by preclinical evidence suggesting that PACAP, but not VIP [35] sensitizes trigeminal neurons, an effect that was blocked by PAC_1_ antagonism.

In support of an emerging role for PACAP signalling in headaches PACAP-38 concentrations have been shown to be elevated during migraine attacks [36, 37] and decreased interictally in episodic cluster headache, with subsequent increases in bout [38]. With an increased genetic understanding of migraine and the identification of multiple susceptibility loci [39], it is somewhat surprising that linkages to novel effective pharmacological targets such as CGRP [40–42] or its receptor are not identified. As such it is less surprising that there is no identified association between PACAP or PAC_1_ signalling in migraine. In comparison, a genome-wide association has been demonstrated for PACAP in cluster headache [43].

### Sleep and circadian rhythms in headache

The interaction between sleep and headache has gained considerable interest due to a strong but complex clinical relationship. This is evidenced from clinical and population studies demonstrating a high penetrance of sleep problems or manifest sleep disorders in headache [44] and an ever increasing number of sophisticated sleep studies [45–47] that point to several major points of physiological and neuroanatomical overlap (for review see [9, 28]).

In agreement with a role for sleep disruption in headaches cluster headache (CH) patients complain of reduced sleep amount – that is complicated by the presence of consistent nocturnal attacks that may directly disrupt sleep. However; CH patients demonstrate poor sleep quality both in- and outside of active cluster bouts [10] highlighting a potential underlying disruption of sleep homeostatic regulatory mechanisms separate from the influence of the nocturnal attacks. This is further supported by a high prevalence of sleep apnea [46], confounded by several overlapping risk factors – e.g. male gender, high body mass index, smoking, and specific sleep-linked attack forms, including hypnic headache [48].

The chronobiological nature of several headaches further highlights a key circadian/circannual component to attack onset, whilst ultradian components have not been widely explored. The most prominent rhythmical headache disorder is CH with its clear circadian [10–12] (commonly during the early night) and circannual periodicity - peak bout incidence potentially related to summer and winter solstice [49]_._ This is the time of year when the difference between night and day is greatest, and in a modern setting, perhaps places the greatest stress on homeostatic entrainment mechanisms. Therefore, it could be postulated that a suboptimal functioning of the gain control in the light-governed entrainment system could induce dysfunctional hypothalamic homeostatic mechanisms [3], leading in turn to increased attack propensity. Migraine on the other hand is most commonly reported to initiate in the early hours of the morning [50] with evidence of a circannual periodicity linked to the light season with fewer attacks during the dark season [51]. This would suggest that CH attacks largely initiate during the early hours of sleep occurring in two common phases – associated with altered environmental light levels and migraine attacks largely initiate during the last hours of sleep/early in the arousal phase occurring most commonly in a single phase – associated with higher environmental light levels. It has been suggested that such nocturnal headache attacks are linked to specific macro-sleep phenomena [52]. While this has not been completely refuted, evidence is limited [45, 47] and recent research has suggested that nocturnal attacks may be linked to the cycling between sleep stages, and not to a particular stage itself [10]. This theory of heightened attack susceptibility during the transitioning from one state to another may give important clues as to the potential mechanisms that underlie attack initiation. For example, the presence of excessive yawning [53] during migraine premonitory symptoms points to a potential excess dopaminergic tone [54]; however, the subsequent transition to headache would be more likely associated with a decreased dopaminergic tone – as dopamine has been shown to be anti-nociceptive at least at the level of the trigeminocervical complex [55, 56].

Traditionally, and due to technical limitations, encephalographic analysis of sleep has been limited to macrostrutural analysis of stage composition. However, increasingly sophisticated analysis methods have revealed changes in the microstructure of sleep. Such analysis of sleep has revealed some interesting changes in headache patients including migraine and CH. Arousals are abrupt changes in EEG frequency of less than 3 s duration. Such arousal phenomena are a part of normal sleep and an increasing number are seen with age. They indicate cortical activation and are generated by systems in the basal forebrain, thalamus, hypothalamus and brainstem via ascending projections. In a population especially prone to poor sleep quality one would expect a high number of arousals, however, counterintuitively, in both migraine and cluster headache a reduced number of arousals have been found [45, 57–59], suggesting that dysfunctional CNS neural networks including hypothalamic, thalamic and brainstem nuclei may be a common feature.

### PACAP in the regulation of sleep

As discussed above the ability to adapt to external environmental conditions is a fundamental principle for the survival of an organism. This allows for seasonal variations in physiology and behavior that optimize our interactions with the local environment. Additionally, as the human intrinsic (“free-running”) circadian period is 24.1 h [60] the ability to entrain the “master clock” in the hypothalamic suprachiasmatic nucleus (SCN) to seasonal light-dark cycles ensures alignment to the astronomical day. The SCN in turn acts as the central circadian regulator ensuring that peripheral oscillators (“local clocks”) regulating local cellular rhythms are synchronized in part via regulation of specific brain circuits [61]. This includes the regulation of the autonomic nervous system [62] that controls peripheral tissue and the rhythmic release of hormones including melatonin from the pineal gland [63] that both entrains local oscillators and inhibits SCN neuronal activity [64] in a negative feedback manner.

Under normal conditions the rhythm of the SCN is primarily influenced by light-dark cycles, with light acting as the prominent “zeitgeber” in both diurnal and nocturnal animals. While common photoreceptors such as rods and cones are involved in light-entrainment non-image forming intrinsically photosensitive retinal ganglion cells (ipRGCs) that express melanopsin encoded by the Opn4 gene play a prominent role [65]. In general, direct projections from light-responsive ipRGCs synapse on SCN neurons giving rise to the retinohypothalamic tract (RHT), with additional sparse projections to other hypothalamic nuclei. Additionally, indirect projections exist via the thalamic intergeniculate leaflet that receives light-sensitive inputs and sends neuropeptide Y projections to the SCN. Early studies in rodents highlighted the presence of PACAP immunoreactivity in a subset of RHT retinal ganglion cells that were responsive to light and projected to the SCN [66]. Later these PACAP containing neurons were shown to express melanopsin and while glutamate has been proposed as the main neurotransmitter in the RHT the role of PACAP is an interesting issue with respect to headache disorders.

Peripherally administered PACAP is an established experimental tool for the induction of migraine [33]. Both PACAP-38 and PACAP-27 potentially cross the blood brain barrier (BBB) in a saturable and non-saturable manner respectively [67, 68], although this is not supported by human studies [32]. The pineal gland lies outside the BBB and is innervated with PACAP immunoreactive fibres that may in part arise from the trigeminal ganglion [69]. Within the pineal gland but not the pituitary PACAP levels show a circadian expression [70] that is phase dependent – with the highest levels occurring during the dark phase in rats. Given that PACAP can stimulate melatonin synthesis [71, 72] and the lack of a functional BBB, intravenous PACAP could, at least in theory, modulate sleep-wake cycles via a direct action on melatonin release. In agreement PACAP administration in rats increased the duration of REM sleep [73]; however, PACAP-38 [74] administration in healthy controls had no impact on the time spent in each sleep stage, but did modulate slow wave sleep. The inconsistency between the current clinical and preclinical data in response to PACAP administration is complex, given likely differences in BBB penetrability and the known dose-dependent opposing actions of PACAP on the SCN.

### PACAP in the SCN

Circadian phases are regulated at the level of the SCN by cell-autonomous, transcription translation feedback loops, whereby Period and Cryptochrome gene expression is inhibited by their respective proteins. The RHT sends light-sensitive projections to multiple regions of the SCN [75] that signal via glutamate [76], aspartate [77] and PACAP [18], as well as indirect projections that utilize NPY [78] and GABA [79] as the key neurotransmitters (for review see [61]). Electrical stimulation of the RHT releases glutamate [80] that induces phase responses and inhibition of glutamatergic signaling blocks SCN responses to light pulses [81] establishing glutamate as the prominent RHT – SCN neurotransmitter. While direct evidence for PACAP release in the SCN is limited, local application of PACAP [66, 82] in-vitro was shown to phase advance SCN neurons during the subjective day via a PAC_1_ dependent mechanism, but not at night, suggesting a role for PACAP in daytime regulation of the circadian cycle. During the subjective late night co-administration of PACAP and glutamate blocks the normal response to glutamate [83, 84], while inhibition of PACAP signalling modulates this response, that was supported by the ability of PACAP to potentiate glutamate induced light responses in-vivo [83]. Conversely, during the early night PACAP potentiated glutamate induced phase delays that was inhibited by blocking PACAP signalling [83]. As such PACAP may act to provide a gain control mechanism for glutamate induced phase shifts that could have a significant determinant on multiple downstream peripheral oscillators [85]. This role of PACAP is further supported by the use of available PACAP or PAC_1_ knock-out mice that maintain a stable activity-rest pattern during constant darkness and demonstrate stable expression of clock genes. Despite this apparently normal circadian phenotype PAC_1_ deficient mice demonstrate impaired photic entrainment in agreement with the above pharmacological data [86, 87] and disrupted circadian food anticipatory behaviours [88].

The proposed mechanism of PACAP and glutamate induced phase alterations is via the light sensitive clock genes, *Period 1* (*per1*) and *Period 2* (*per2*) [84]. Ex-vivo glutamate administration on SCN brain slices induces robust increases in *per1* and *per2* expressions; however, micromolar concentrations of PACAP alone was unable to modulate their expression. In agreement with a role for PACAP as a modulator, pre-administration of micromolar concentrations of PACAP completely blocked the effect of glutamate, whilst nanomolar concentrations induced *per1* and *per2* expression [84]. While data on the role of specific clock genes in headache are limited the recent discovery of human mutation in the catalytic domain of the gene encoding casein kinase 1δ (CK1δ; CK1δ-T44A) that was associated with both familial advanced sleep phase syndrome (FASPS) and migraine with aura [89]. Importantly phosphorylation of PER proteins by CK1 proteins regulates the speed of the circadian clock [90]. PER1 and PER2 are phosphorylated at multiple sites by CK1δ and CK1ε that facilitates their degradation and subsequent release of inhibitory repression of Clock/BMAL1 as key elements of the cell-autonomous transcription translation feedback loops [91, 92]. Thus this loss of function mutation that co-expresses altered circadian phases and migraine with aura indirectly highlight a potential relationship between PER2 regulation and migraine. With respect to CH several studies have explored potential relationships with clock gene variants due to the striking circadian and circannual periodicity of attacks. While no association has been found between CH and *per3* or the T-C Clock gene polymorphism [93, 94] a recent publication determined a potential association between the rs12649507 Clock gene polymorphism [95] that has been previously associated with sleep duration [96]. Patients with the rs12649507 AA genotype additionally demonstrated increased Clock gene expression, raising the possibility that CH may result from circadian misalignment.

The effects of administration of PACAP on sleep in humans has not been extensively studied and the PACAP-effects observed in animals (increase in REM-sleep) [97] have so far not been reproduced in humans under the described conditions. This does not exclude an effect in humans however as there are many variables that could be changed. As noted previously, a recent study has implicated a common variant of the PACAP receptor gene (ADCYAP1R1) [43] in CH but the results were not replicated in a larger study [98]. Further, the specifics of how systemically administered PACAP could regulate circadian rhythms remains to be elucidated.

## Conclusion

PACAP is emerging as an important molecular target in the pathophysiology of primary headache disorders, with a particular focus on migraine and CH. It is well established that there is a clear clinical association between these conditions and sleep disturbances; while preclinical studies are beginning to propose novel mechanisms that underlie these shared etiologies [9, 89]. It is clear that migraine [50] and CH [10–12] have a clear rhythmicity, both at the circadian and circannual level and as such future research should explore both the underlying mechanisms of this association and the potential for novel translational lifestyle and pharmacological targets to lighten the burden of disease.

There is a need to develop a greater understanding of the rhythmic changes observed in headaches. For example, while PACAP and other molecules such as CGRP and nitroglycerin can be potent migraine triggers, little is known about circadian and circannual variability in their response. Experimentally, individual aspects of circadian variation in trigeminovascular nociceptive processing, sleep and autonomic regulation may be studied but it is ultimately in the combination of our knowledge of these functions that true progress can be made. Additionally, while the hypothalamus is emerging as a key modulator of several primary headache conditions, with respect to circadian and circannual periodicity we should not lose sight of the role of peripheral oscillators “local clocks”. The successful integration of light entrainment to direct biological function lies not only with the SCN, but also its alignment with local oscillators. Finally, the described effect of PACAP-administration on sleep needs to be studied in headache patients specifically.

## Abbreviations

BBB: Blood Brain Barrier
BF: Basal Forebrain
CH: Cluster Headache
CK1δ: Casein Kinase 1 delta
DR: Dorsal raphe
FASPS: Familial advanced sleep phase syndrome
ipRGCs: Intrinsically Photosensitive Retinal Ganglion Cells
LC: Locus Coeruleus
LDT: Laterodorsal Tegmental Nuclei
LH: Lateral Hypothalamus
NREM: Non-Rapid Eye Movement
PACAP: Pituitary Adenylate Cyclase-Activating Peptide
PB: Parabrachial
Per: Period
PPT: Pedunculopontine
REM: Rapid Eye Movement
RHT: Retinohypothalamic Tract
SCN: Suprachiasmatic Nucleus
TMN: Tuberomammillary Nucleus
VLPO: Ventrolateral Preoptic Area
vPAG: Ventral Periaqueductal Grey
